# High Resolution Mass Profile of Bufadienolides and Peptides Combing with Anti-Tumor Cell Screening and Multivariate Analysis for the Quality Evaluation of Bufonis Venenum

**DOI:** 10.3390/molecules24101943

**Published:** 2019-05-20

**Authors:** Rongrong He, Hongyue Ma, Jing Zhou, Zhenhua Zhu, Xiang Lv, Quan Li, Hengbin Wang, Yanqing Yan, Niancui Luo, Liuqing Di, Qinan Wu, Jinao Duan

**Affiliations:** 1 Jiangsu Collaborative Innovation Center of Chinese Medicinal Resources Industrialization, and Jiangsu Key Laboratory for High Technology Research of TCM Formulae, College of Pharmacy, Nanjing University of Chinese Medicine, Nanjing 210023, China; herongrongan@163.com (R.H.); 04040416@163.com (Z.Z.); lyxhj09@163.com (X.L.); diliuqing928@163.com (L.D.); qnwyjs@163.com (Q.W.); duanja@163.com (J.D.); 2 Leiyunshang Pharmaceutical Company Ltd., Suzhou 215003, China; 18969503503@163.com (Q.L.); Whb2002y@163.com (H.W.); 17835424756@163.com (Y.Y.); niancuiluo000@126.com (N.L.)

**Keywords:** Bufonis Venenum, quality evaluation, bufadienolides, peptides, pre-fractionation mass spectrometry profile

## Abstract

In order to evaluate the quality of Bufonis Venenum commercial herbs, a three-step qualitative and quantitative research study was performed. Firstly, we tried to identify small molecules and peptides in Bufonis Venenum using pre-fractionation chromatography and high-resolution mass spectrometry. The database search of the small molecules and peptides of Bufonis Venenum revealed that the dried venom consisted of free/conjugated-type bufadienolides and peptides with a mass range of 0.4–2 kDa. Secondly, we used partial least squares (PLS) multivariate statistical analysis to screen bufadienolides markers (VIP > 1.5) responsible for the anti-tumor cell activity of Bufonis Venenum, including 21 identified bufadienolides and 7 unknown compounds. It is noticeable that these bufadienolide markers could not be recognized by traditional HPLC-UV based spectrum-effect relationship analysis (correlation coefficient ranging from −0.24 to 0.40). Finally, we proposed a weight coefficient-based corrected total contents of 9 bufadienolides as a quality evaluation indicator, which had good correlation with inhibitory effects on tumor cells of commercial Bufonis Venenum. The correlation coefficient increased from 0.4 to 0.6. Thus, our pre-fractionation chromatography and mass spectrometry strategy had significant advancement over the traditional spectrum–effect relationship method for chemical marker identification. These results could be crucial and helpful in the development of a quality evaluation method that could reflect the pharmacological activity of Bufonis Venenum.

## 1. Introduction

In recent years, Chinese medicine has made significant progress in modernization and globalization, however, the quality evaluation of TCM (traditional Chinese medicine) is still in a relatively simple stage. The main technical methods to control the quality are still based on traditional traits, microscopic identification and, physical and chemical testing, supplemented by the corresponding content determination [1,2,3]. The development of multi-dimensional chromatography and LC/MS technology has enabled the qualitative identification and quantitative detection of hundreds of compounds from a single herb [4,5,6] and even qualitative identification or quantitative evaluation of an herb from different TCM formula preparations [7,8,9]. The detection methods mentioned above only use the content of one or several components in the medicine as the indicator of quality evaluation. However, the composition of traditional Chinese medicines is complex and diverse. This method of chemical evaluation does not have specific properties because the “indicative component” of most Chinese medicines is neither its exclusive component nor its active ingredient, while the inherent “multi-component and multi-target” characteristics of Chinese medicine require the establishment of a unique quality and biological activity evaluation system, which is different from chemistry medicine [10,11]. The quality evaluation method of Atractylodis macrocephalae rhizome, a traditional Chinese medicine, is TLC identification and determination of extract content; while the former is limited to qualitative research, the latter is not specific. Both quality assessment methods can not accurately reflect its relevance to clinical efficacy because Atractylodis macrocephalae rhizome mainly contains volatile oils, lactones, polysaccharides, and amino acids, but the volatile oils and lactones are the main medicinal ingredients [12,13].

The multi-component and multi-target characteristics of traditional Chinese medicine pose great challenges for its quality analysis and safety evaluation. Traditional analytical techniques are not yet fully reflective of their clinical efficacy and safety. Metabolomics technology has the characteristics of high throughput, high resolution, and high sensitivity, and has unique advantages in the quality identification of traditional Chinese medicine [14,15,16]. The modernization research of traditional Chinese medicine based on metabolomics technology is in line with the theory of the overall view of the drug quality basis [17,18]. It is easy to clarify the multi-target scientific connotation of traditional Chinese medicines on complex biological systems and also to show systematic advantages in the evaluation of Chinese medicine quality and safety.

Bufonis Venenum is the dry secretion of the posterior glands and skin of the *Bufo bufo gargarizans* Cantor and *Bufo melanostictus* Schneider. The main metabolites of Bufonis Venenum include small molecules and macromolecular compounds. The small molecule metabolites mainly include bufadienolides, alkaloids, sterols, and other compounds [19,20,21] and the macromolecular constituents are mainly polypeptides and proteins [22,23]. The bufadienolides are classified into free compounds and conjugated compounds. Among them, the free compounds are further classified into five categories according to the substituents on the skeletons, including bufalin (A_1_), resibufogenin (A_2_), arenobufagin (A_3_), psi-bufarenogin (A_4_), and epoxy resibufogenin (A_5_) [24]. The current Chinese Pharmacopoeia only has clear requirements and standards for the content of resibufogenin and cinobufagin, which does not provide complete chemical information for quality assessment [25,26,27] and ignores the content and efficacy of other important compounds. For example, an MTT assay showed that the inhibition rate of resibufogenin, even at higher contents, was lower on human cancer cells than other bufadienolides [28]. Moreover, the Traditional Chinese Patent Medicine, Huachansu, which has low contents of bufadienolides and high levels of peptides, is clinically used as an anti-tumor drug. Therefore, the content of a single chemical component is unscientific as an indicator for evaluating the quality of TCM. More reliable analytical methods are needed to comprehensively analyze the active compounds and peptides of Bufonis Venenum. This study established a quality evaluation method, based on metabolomics and peptidomics, to identify the active markers for quality evaluation of Bufonis Venenum.

## 2. Results and Discussion

### 2.1. Qualitative and Relative Quantitative Analysis of Small Molecues in Pre-Fractionated Bufonis Venenum by LC-HMRS

**Figure 1:**
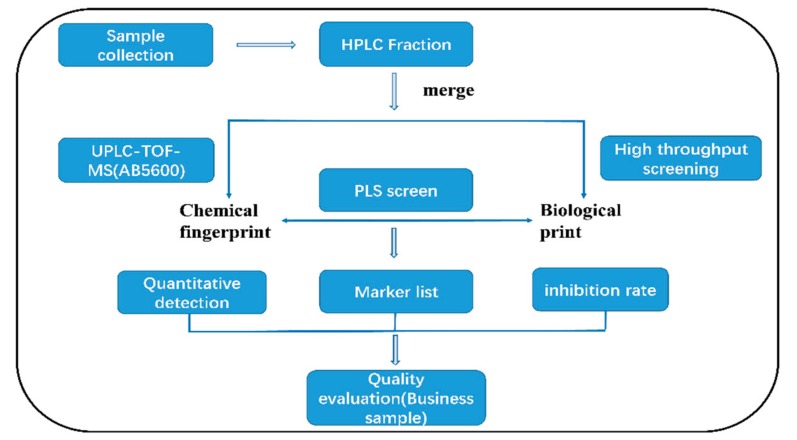
Pipeline for chemical marker discovery and application to the quality evaluation of commercial Bufonis Venenum.

**Figure 2:**
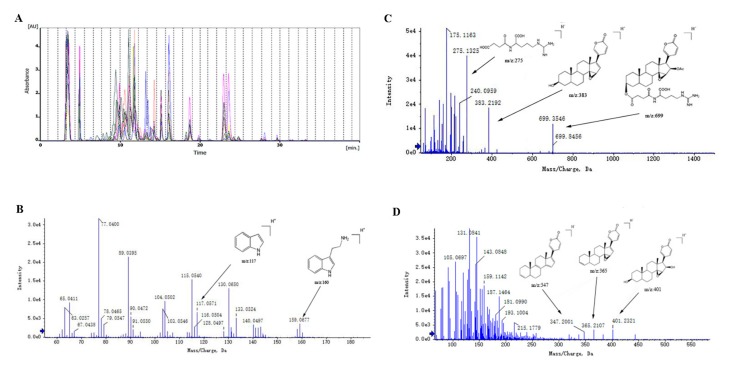
(**A**) HPLC fractions from heterogeneous Bufonis Venenum were used for the marker discovery. The fractions were subsequently quantitatively or semi-quantitatively measured by CID- MS/MS. Identification of Indole-3-acetaldehyde (**B**), 3-(N-suberoyl argininyl)bufalin (**C**), and cinobufagin (**D**), based on MS/MS spectrum of the ion at *m*/*z* 160.05757, *m*/*z* 699.3966, and *m*/*z* 443.2421 in positive mode.

**Figure 3:**
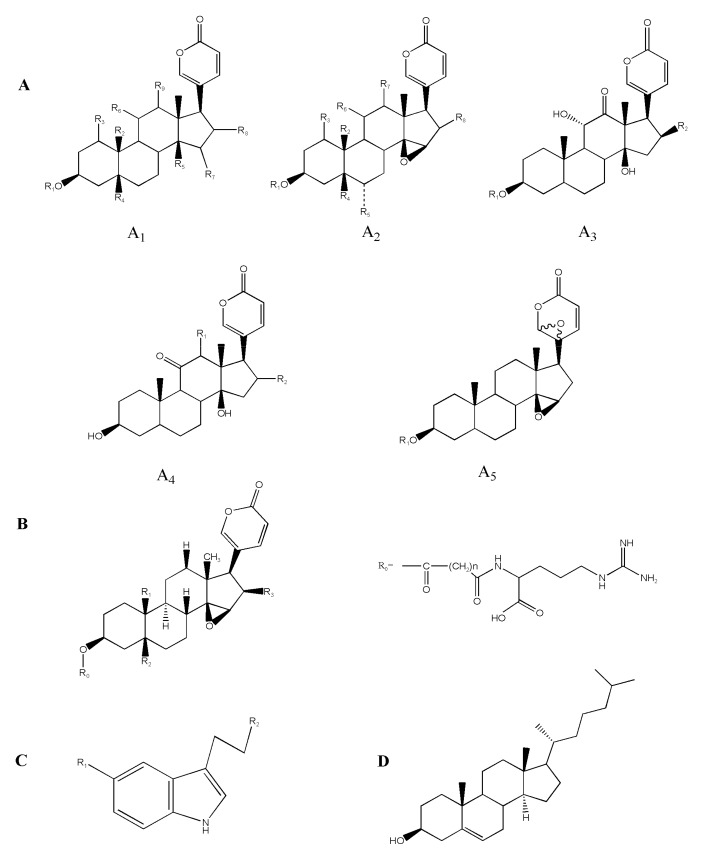
Skeletons of chemical constituents in Bufonis Venenum; (**A1**) bufalin, (**A2**) resibufogenin, (**A3**) arenobufagin, (**A4**) psi-bufarenogin, (**A5**) epoxy resibufogenin, and (**B**) conjugated bufadienolides, (**C**) indole alkaloid, and (**D**) cholesterol.

This study was carried out according to the flow chart shown in Figure 1. The one-dimensional liquid phase separation was performed to generate 180 fractions from 9 Bufonis Venenum products (Figure 2A). All fractions were further analyzed by high-resolution mass spectrometry (HRMS) (AB Sciex 5600 triple TOF) to detect the presence of secondary metabolites. Thus, a series of steroids, indole alkaloids, and other metabolites were annotated. The MS/MS fragmentation of these constituents is shown in Figure 2. For example, the [M + H]^+^ ion at *m*/*z* 160.07 was identified as a tryptophan compound, Indole-3-acetaldehyde (Figure 2B). Its MS^2^ ion at *m*/*z* 117.05 is a [M+H-(CH_2_)_2_NH_2_]^+^ ion. Figure 2C,D are two kinds of bufadienolides. The [M + H]^+^ ion at *m*/*z* 699.84 was identified as the conjugated bufadienolide 3-(N-suberoyl argininyl) bufalin (Figure 2C). Its MS^2^ ion at *m*/*z* 383.2 was assigned to the steroid skeleton of cinobufagin and the [C_10_H_18_O_5_N_4_ + H]^+^ ion at *m*/*z* 275.1 was assigned to the succinyl argininyl ester side chain. The [M + H]^+^ ion at *m*/*z* 443 was identified as the free bufadienolide cinobufagin (Figure 2D) and its MS^2^ ion included the following ions: [M+H-CH_3_CHO]^+^ at *m*/*z* 401.2, [M+H-CH_3_COOH-H_2_O]^+^ at *m*/*z* 365.2, and [M+H-CH_3_COOH-2H_2_O]^+^ at *m*/*z* 347.2. The main components and skeletons of chemical constituents in Bufonis Venenum are shown in Figure 3 and Table S1, including the following five free bufadienolides: Bufalin (Figure 3(A1)), resibufogenin (Figure 3(A2)), arenobufagin (Figure 3(A3)), psi-bufarenogin (Figure 3(A4)), epoxy resibufogenin (Figure 3(A5)), conjugated bufadienolides (Figure 3B), indole alkaloid (Figure 3C), and cholesterol (Figure 3D).

The intensity of each precursor detected by HMRS could be used for the relative quantitation. This label-free quantification approach allow relative metabolite abundances to be compared in all fractions. The metabolites dynamics could be characterized in different Bufonis Venenum products.

### 2.2. Qualitative Analysis of Peptides in Bufonis Venenum

**Figure 4:**
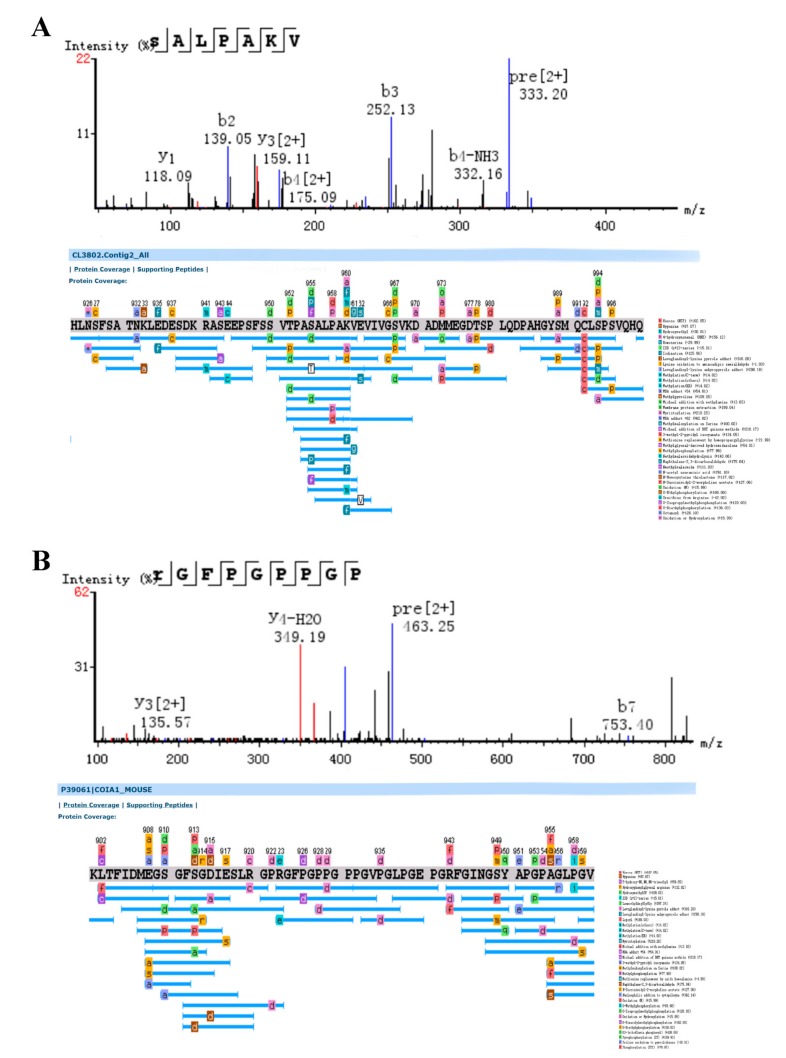
The MS/MS spectrum of the best unique peptide and the peptide spectrum matches, indicated by blue lines below the sequences of precursor protein. The best peptide sequences matched to CL3802 (**A**) and P39061 (**B**) are SALPAKV and RGFPGPPGP.

Mass spectrometry raw data were analyzed for the identification of peptides in Bufonis Venenum. De novo analysis, database, and homology searches with manual validations showed some peptides, which is composed of 261 features, with masses ranging from 0.4–2 kDa. They were derived from 42 proteins, identified using the PEAKS software with acquired MS and MS/MS spectra against the house-built protein database using de novo RNA sequencing. The 10 ions in Table 1 presented main clusters of masses around 0.9 kDa, with precursor ion charges ranging from 2 to 3. Figure 4 is MS/MS spectrum of the best unique peptide and peptide sequence matched with their corresponding proteins in Bufonis Venenum. Usually there are three kinds of poly-peptide skeletons on the general fracture mode, the a/b/c are on behalf of the N-terminal and x/y/z represent the C-terminal of peptide. The best peptide sequences matched to CL3802 (Figure 4A) and P39061 (Figure 4B) are SALPAKV and RGFPGPPGP. The 4th amino acid from the left in A is Pro, which can be verified by the difference in *m*/*z* between ion b4 and b3. 

The MTT screen showed that peptides in the Bufonis Venenum inhibited tumor cells at the μg/mL level, but the bufadienolides have more significant inhibition at the ng/mL level. That suggested that peptides might have a weak contribution to the anti-tumor activity of Bufonis Venenum. However, the recent study showed that the peptides had a strong analgesic activity [29]. They could be key constituents for the pain-relieving effects of Bufonis Venenum. Thus, these peptides should be subject to additional study in the development of quality evaluation testing of Bufonis Venenum.

### 2.3. Discovery of Chemical Markers for the Anti-Tumor Cell Effects of Bufonis Venenum

**Figure 5:**
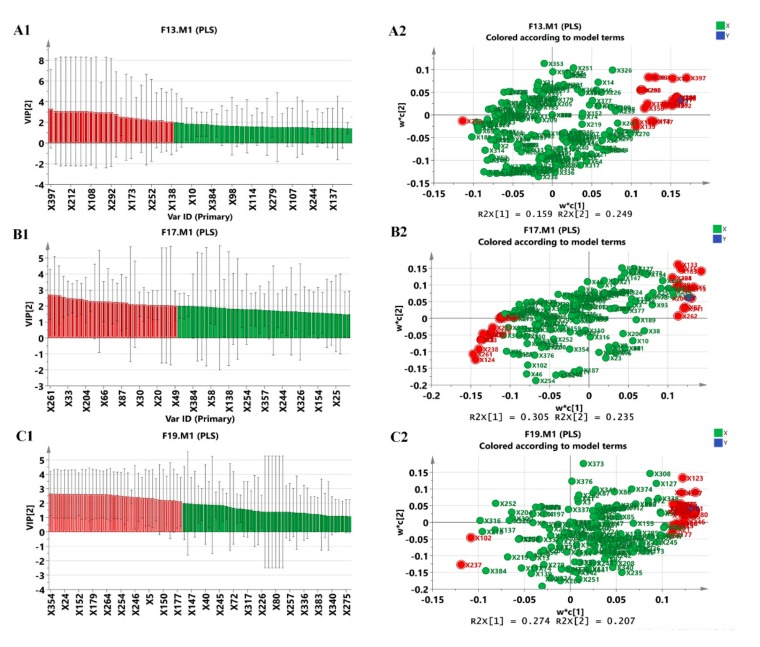
Candidate markers were screened by PLS analysis using SIMCA-P software. The ions marked in red with VIP > 1.5 (**A1**–**C1**) are selected as the chemical markers better relevant to the inhibition of tumor cells.

MTT results were used as independent variables (Y) and the intensities of all precursor ions were used as X for the partial least squares (PLS) analysis (Figure 5). PLS is a multivariate statistical approach used for variable reduction and separation into class. S-plots were calculated to visualize the relationship between covariance and correlation within the PLS results. Variables (X_1−n_) that had significant contributions to biological activity (Y) were considered as potential biomarkers. The higher the VIP (variablesp importance of projection) value means the higher the correlation and contribution to the anti-tumor cell activity. Figure 5 shows the red plots with a VIP value greater than 1.5, positively correlated with the variable Y in the same quadrant and negatively correlated in the diagonal quadrant. Here, the constituents with an ion intensity greater than 1000 and a VIP value greater than 1.5 were selected as markers for the following quality evaluation of Bufonis Venenum. The potential precursor ions in Figure 5(A2) (R2X[1] = 0.159, R2X[2] = 0.249) and C2 (R2X[1] = 0.274, R2X[2] = 0.207) which are consistent with the screening index are positively correlated with the variable Y. Some of the precursor ions in Figure 5(B2) (R2X[1] = 0.305, R2X[2] = 0.235) are negatively correlated with the variable Y. It was observed that bufadienolides had a higher VIP value than bufotenines and peptides responsible for anti-tumor cell activity. Thus, we screened 28 (X1–X28) chemical markers (Table 2) from approximately 500 constituents, including 17 bufadienolides, and some unidentified constituents. 

Considering the great challenges and difficulties for the quantitation analysis of peptides using common HPLC-UV technology, it is not suitable to use peptides as quality evaluation markers of Bufonis Venenum in industry. So, these 9 bufadienolides (resibufogenin, cinobufagin, bufalin, arenobufagin, desacety-bufotalin, gamabufotalin, cinobufotalin, bufotalin, and telocinobufagin) with good anti-tumor cell activity are easily obtained as chemical markers for the subsequent quality evaluation. It can be seen from Table 2 that the VIP values of these nine markers were all above 2.0 and their corresponding intensity was high.

**Figure 6:**
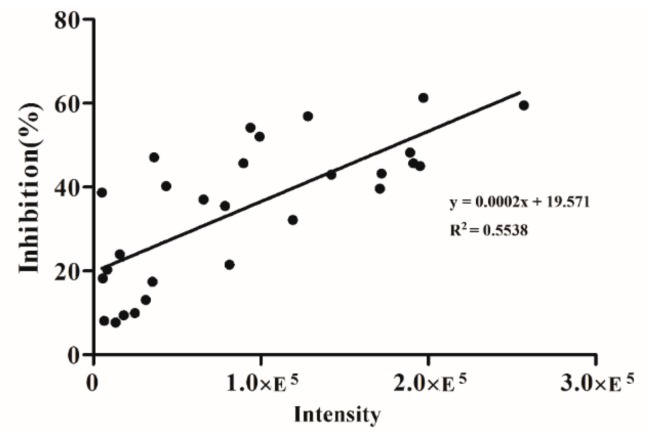
The correlation between the ion intensity of the candidate chemical markers at all fractions and their inhibition of tumor cells (*p* < 0.01).

In addition, according to the inhibition effect of the Bufonis Venenum on tumor cells, the mass spectrometry intensity of the markers across different fractions was linearly analyzed. The analysis results are shown in Figure 6. The overall linear relationship between the ion intensity of these markers and the anti-tumor cell activity is good (*p* < 0.01) and higher mass spectral intensities correspond to higher biological activities.

### 2.4. Classic Spectrum-Effect Relationship Analysis on the 9 Bufadienolide Markers in Different Batches of Bufonis Venenum Products

The spectrum-effect relationship was considered to be a crucial method for the QC markers’ discovery of TCM. In the classic spectrum-effect relationship study, chromatographic peaks or constituents linking with bioactivity were identified for the quality evaluation of TCM [30]. However, this method may not be accurate, due to the interference of complex components in non-fractionated crude extracts to the bioassay. So, we tried to compare the usefulness for the identification of chemical markers using a classic spectrum-effect relationship method with our pre-fractionation mass strategy. Here, the spectrum–effect relationships between HPLC fingerprints of 9 bufadienolides and the anti-tumor cell activities of different batches of Bufonis Venenum were investigated using correlation analysis. Firstly, we applied a common HPLC-UV industry approach to develop quantitative assays to measure 9 bufadienolide markers in Bufonis Venenum. The linear relationship of the methodological investigation is shown in Table 3. All the calibration curves showed a good linearity, with correlation coefficients (r2) no less than 0.999. The corresponding concentration at the signal-to-noise ratio (*S*/*N*) of 3 and 10 is the LOD (limit of detection) value and the LOQ (limit of quantity) value. The intra-day and inter-day precision, repeatability, stability, and recovery results are shown in Table 4 below. All results show that the analytical method is accurate and feasible.

**Figure 7:**
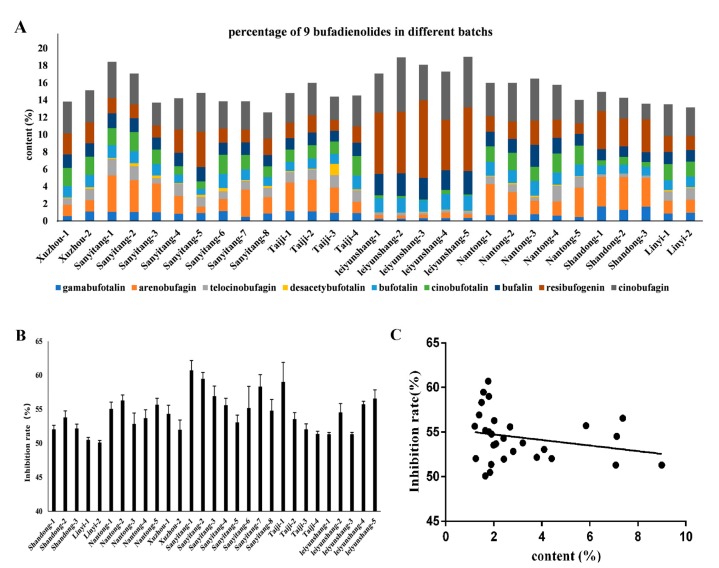
Percentages of 9 bufadienolides in different batches of Bufonis Venenum products, determined by HPLC-UV (**A**); inhibition rate of different batches of Bufonis Venenums on SMMC-7721 tumor cells (**B**); and the correlation between the antitumor cell activity of 29 batches of Bufonis Venenum and the content of resibufogenin (**C**).

Next, this assay was applied to 29 batches of Bufonis Venenum products (the sample list is shown in Table S2). The contents of 9 bufadienolide markers in these commercial batches are shown in Figure 7A. The anti-tumor cell activity of these Bufonis Venenums are shown in Figure 7B. Pearson correlation analysis was used for the spectrum–effect relationships between the area values of 9 bufadienolides in the HPLC-UV fingerprints and the values of anti-tumor cell activities. Their correlation coefficients were all below 0.4, ranging from −0.24 to 0.40. The correlation between resibufogenin and antitumor cell activity is shown in Figure 7C and the results of correlation analysis of the remaining bufadienolides with antitumor cell activity are shown in Table 5. This clearly demonstrated that the anti-tumor cell activity of 29 batches of Bufonis Venenums had no correlation with the 9 bufadienolide peaks in the UPLC-UV fingerprints.

So, this classic spectrum-effect relationship study isn’t well recognized in bufadienolide markers in Bufonis Venenums. The potential reason for this may be the interference of multiple components in the bioassay and the difference in tumor cell inhibition of the 9 bufadienolides. Thus, our pre-fractionation mass strategy was a significant advancement over the traditional spectrum–effect relationship method. 

### 2.5. Multivariate Analysis of 9 Bufadienolide Marker Content in Commercial Bufonis Venenums

The total contents of 9 bufadienolides in Bufonis Venenum ranged from 11.63–19.02% (Figure 7A). Resibufogenin, cinobufagin, and arenobufagin were the major components, with average ratios of 2.96%, 3.62%, and 2.14% in Bufonis Venenum. We observed that the content of resibufogenin in leiyunshang is significantly higher than the other samples, followed by samples from Nantong, Jiangsu Province. Interesting, Leiyunshang samples have a high resibufogenin content and a low arenobufagin content, but other samples had a low resibufogenin conten and a high level of arenobufagin. So, the relative ratios about resibufogenin/arenobufagin could be used to clarify the different origins of Bufonis Venenum products.

**Figure 8:**
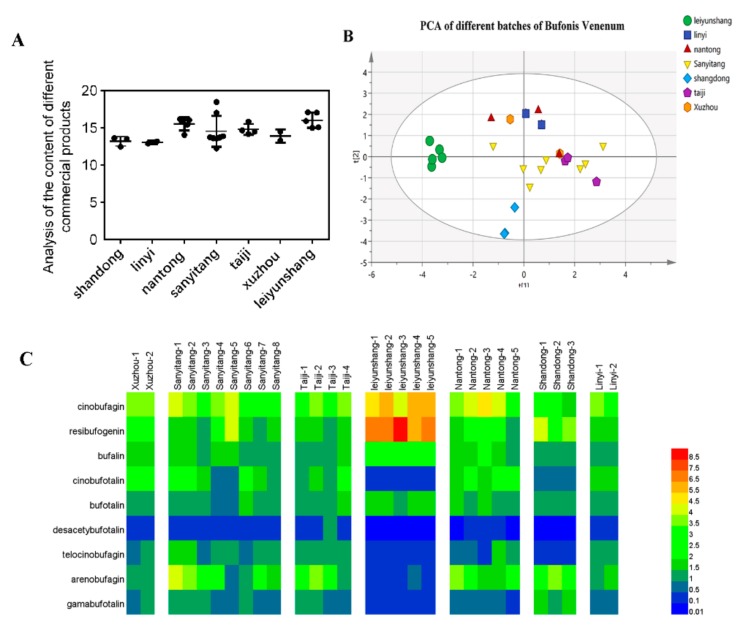
Analysis of the content of different commercial products (**A**); Principal component analysis (**B**) and Heat map (**C**) showing the contents of 9 bufadienolides and the varied chemical quality of different batches.

We further used a multivariate data analysis method to show significantly different chemical phenotypes in different origins of Bufonis Venenums. The content detection data of 9 bufadienolides were transferred to SIMCA-P software for principal component analysis (PCA). PCA is an unsupervised multivariate statistical approach used for variable reduction and separation into classes. As illustrated in the PCA score plot (R2X[1] = 0.461, R2X[2] = 0.2) and heat map (Figure 8), their chemical quality varied with different regions. In principle, the Bufonis Venenum were clustered into several categories according to different origins in the plot, revealing inter-group differences in the chemical phenotype. On the other hand, the Bufonis Venenum in each group (within the same region) were obviously dispersed in the PCA score plots, indicating individual intra-group differences in composition. It was observed that samples from the Leiyun Shang Pharmaceutical Base can be completely separated from samples from other commercial batches of Bufonis Venenum. These results clearly showed that the chemical quality of Bufonis Venenum is substantially varied at the batch and region levels. 

### 2.6. Correlation Between the Bufadienolide Marker Contents Corrected by the Weight Coefficient and Tumor Cell Inhibitory Effects

On the basis of the contents of total bufadienolides in Bufonis Venenum and the inhibitory effects on tumor cells SMMC-7721 of Bufonis Venenum, the linearity correlation was analyzed using the data from commercial batches. A poor linearity was observed between the content of total bufadienolides and the inhibitory effects on tumor cells. This mainly resulted from the difference in tumor cell inhibition of the 9 bufadienolides (Table S3). For example, resibufogenin has a high content in all samples (Figure 7A), especially in the Leiyun Shang Pharmaceutical Base. However, its inhibition on tumor cells was the weakest among all the markers tested. So, the high resibufogenin content does not necessarily correspond to a good therapeutic effect.

Considering different markers might have different contributions on the anti-tumor effects of Bufonis Venenum, we used a weighted coefficient to combine multiple marker content into a corrected total content, proposed as follows Equation (1). The weight Equation (2) was determined according to the inhibitive intensity of the 9 bufadienolides on tumor cells and their contribution to the total activity of Bufonis Venenum.

(1) $$S=\sum_{i=1}^{m}\left(A_{i}B_{i}\right)$$

(2) $$B_{i}=\sqrt{x_{i}y_{i}}$$

**Figure 9:**
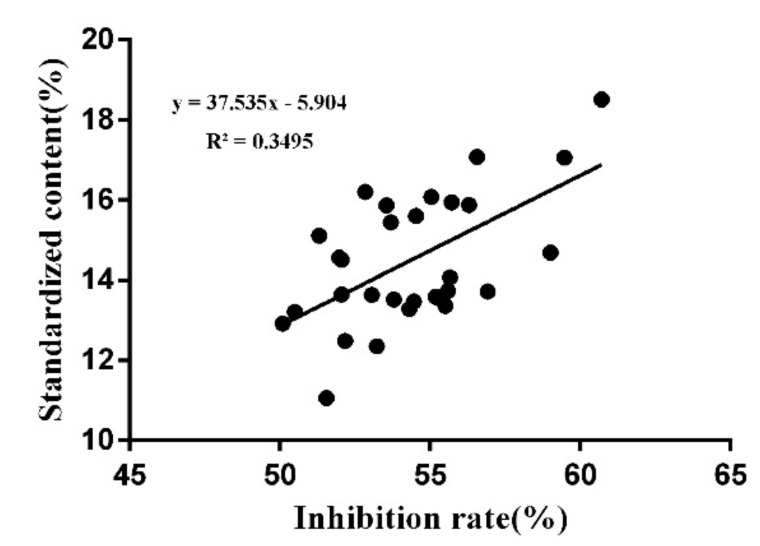
Correlation between the bufadienolide marker contents, corrected by the weight coefficient and tumor cell inhibitory effects (*p* < 0.01).

In the above two formulas, $A_{i}$ was determined as the content of each component, measured in 29 batches of samples. The value $B_{i}$ was the weight coefficient of each component, $x_{i}$ (Table S3) is the standard value for cancer cell inhibition rates, and $y_{i}$ (Table S4) is the standardized value of the VIP value from the PLS analysis. Equation (1) is the calculation method for the weight content of each component in Bufonis Venenum and a new quality evaluation method is established according to this formula. After the correction using the weight coefficient, we found that the corrected total content of 9 bufadienolides, in commercial products, was strongly related with their inhibitive activities on tumor cell SMMC-7721. The correlation coefficient increased from 0.4 (*p* < 0.05) to 0.6 (*p* < 0.01) (Figure 9). Moreover, variety in corrected total contents could reflect batch-to-batch consistency. It can be seen from Figure 8C that the total content difference between batches is large. Among them, the total bufadienolide content in five batches of products from Leiyun Shang Pharmaceutical Base is not only high, but the inter-batch variation of is also the smallest. The above results indicate that the marker contents of products from Leiyun Shang Pharmaceutical Base is high and the inter-batch quality is stable. So, this method can be used for quality evaluation of commercial Bufonis Venenum products.

## 3. Conclusions

We proposed a pipeline to globally identify small molecules and peptides in Bufonis Venenum. The database search of the small molecules and peptides of the Bufonis Venenum revealed that the dried venom consisted of free/conjugated-type bufadienolides and peptides with a mass range of 0.4–2 kDa. Furthermore, we screened 28 chemical markers by using a pre-fractionation high-resolution mass profile combined with an anti-tumor cell assay and PLS multidimensional analysis. Due to the challenges and difficulties in the quantitative analysis of peptides, nine bufadienolides were selected as quality evaluation markers for application in the pharmaceutical industry. Interestingly, we observed that these markers could not be recognized by classic spectrum-effect relationship analysis. Thus, our pre-fractionation mass spectrometry strategy provides a better and more productive method for the identification of quality evaluation markers than the traditional spectrum-effect relationship. Finally, we proposed a weight coefficient-based method to correct the total bufadienolide contents for the quality evaluation of commercial Bufonis Venenum. In summary, these results could be crucial and helpful in the development of a quality evaluation method that can reflect the correlation between the chemical marker content and the pharmacological intensity of Bufonis Venenum.

## 4. Materials and Methods

### 4.1. Standard Solutions and Sample Preparation

Bufonis Venenum were obtained from 9 different provinces, Shandong, Henan, Heilongjiang, Anhui, Tianjin, Hunan, and Guangdong in China. The Bufonis Venenum was combined and dehydrated to a constant weight and stored frozen at −80 °C until use.

A total of 29 batches of Bufonis Venenum were collected from different producing areas, ultrasonically extracted with 90% methanol for 1 h, centrifuged to remove the supernatant, and passed through a microporous membrane for use.

A total of 9 bufadienolide standards were used, including resibufogenin, cinobufagin, bufalin, desacety-bufotalin, arenobufagin, gamabufotalin, cinobufotalin, bufotalin, and telocinobufagin. Each bufadienolide was dosed with methanol to a 1 mg/mL stock solution and diluted to different concentrations (0.4, 1, 4, 10, 20, and 50 μg/mL) for linear investigation.

### 4.2. HPLC Fractionation and Collection of Small Molecule Compounds

One-dimensional liquid phase separation of the methanol extract of Bufonis Venenum was performed using a Durashell RP column (5 μm, 150 Å, 4.6 × 250 mm). Mobile phases A (0.1% formic acid) and B (acetonitrile) were used for gradient elution. The elution gradient is as follows: 0–2 min, 25–32% B; 2–8 min, 32–47% B; 8–16 min, 47–49% B; 16–22 min, 49–54% B; 22–24 min, 54–80% B; 24–29 min, 80% B; 29–30 min, 80–25% B; and 30–40 min, 25% B. The eluent was collected every minute. The 40 HPLC fractions were finally combined into 20 fractions based on the chromatographic peak pattern. All samples were concentrated and evaporated and then reconstituted with 200 μL of 80% methanol, centrifuged at high speed, and the supernatant was taken for qualitative analysis.

### 4.3. Extraction of Peptides

A total of 100 mg Bufonis Venenum were dissolved in 1 mL water and extracted ultrasonically for 30 min. Then, the supernatants (about 1 mL) were transferred onto the center of 10 KD molecular weight cut-off separately. After centrifugation at 8000 r/min for 20 min, the filtrate was combined. Finally, all the samples were desalted by oasis HLB columns and dried by a concentrator at 40 °C.

### 4.4. Mass Spectrometry Analysis

The fractions were re-solubilized and analyzed using LC-MS/MS (RIGOL, Beijing, China). The supernatant was eluted onto the Phenomenex column (Synergi^TM^ 2.5 μm Fusion-RP 100 Å, 50 × 2 mm, Phenomenex, Torrance, CA, USA) at a flow rate of 0.4 mL/min and were analyzed by AB Triple-TOF^TM^ 5600 (AB SCIEX, Framingham, MA, USA). The mobile phase was composed of solvent A (0.1% formic acid in water) and solvent B (acetonitrile). The column was subjected to a linear gradient system. The gradient was as follows: 0–2 min, 20–30% B; 2–6 min, 30–35% B; 6–9 min, 35–60% B; 9–10 min, 60–95% B; 10–11 min, 95% B; 11–11.2 min, 95–20% B; 11.2–13 min, 20% B. The injection volume was 5 μL. The best conditions for mass spectrometry are as follows: Scan mode (positive ion scan); sample cone voltage (30 V); capillary voltage (3000 V); source temperature (120 °C); collision energy (5 eV); desolvation gas flow (600 L/h); and desolvation temperature (300 °C). The mass spectrometry was operated in data-dependent mode, automatically switching between MS and MS2 acquisition. Survey full-scan MS spectra (*m*/*z* 100–1500) were acquired in the AB Sciex 5600 Triple TOF (AB SCIEX). The 20 most intense ions were sequentially isolated and fragmented by collision induced dissociation (CID). Both small molecules with less than +2 and peptides with assigned charge states from +2 to +6 were selected for MS/MS fragmentation. Ten samples were used as one batch. After the start and end of each batch analysis, quality evaluation samples and blank methanol samples were used for quality evaluation to ensure the stability of the analysis process.

### 4.5. Bioinformatic Analysis of Peptide Sequence

Mass spectrometry raw data of peptide fractions were searched in PEAKS Studio (Shanghai Omicsolution Co., Ltd. Shanghai, China) using tolerance of precursor and a fragment mass of 10 ppm and 0.025 Da. De novo peptides, whose average local confidence (ALC) scores ≥80% were selected for database searches against the house-built transcriptome-derived database and an amphibian database downloaded from NCBI with the same conditions. The peptide false discovery rate (FDR) was predictable by the decoy fusion method and was selected at a maximum of 1%. Post-translational modifications and homology searches were performed by PEAKS PTM and SPIDER (two search modes for PEAKS software), respectively. The peptide false discovery rate can be predicted by the decoy fusion method and the maximum selection is 1%. The house-built database derived from the de novo transcriptomics sequence was used for the MS/MS analysis of peptides.

### 4.6. MTT Experiment

The human hepatocarcinma cell lines (SMMC-7721) and human lung adenocarcinoma cell line (A549) (Chinese Academy of Sciences, Shanghai, China) were routinely cultured in L110 high glucose DMEM medium and the cells in the logarithmic growth phase were taken to prepare a cell suspension, which was inoculated into a 96-well plate at 5000 per well, and the periphery was filled with a PBS solution. A total of 200 μL of different concentrations of the drugs were added and a DMEM medium, containing 0.1% methanol in the same volume, was used as a blank control. Additionally, 6 duplicate wells were set for each drug. After this, cells were cultured at 37 °C in a humidified atmosphere of 5 % CO_2_. After 48 h, 20 μL of 3-(4,5-dimethylthiazol-2-yl) 2,5- diphenyltetrazolium bromide (MTT) solution was added to each well and incubation continued for 4 h. The supernatant was removed, 100 μL of 1% DMSO was added to each well to dissolve the crystals, and the absorbance was measured at a wavelength of 490 nm to calculate the cell proliferation inhibition rate.

### 4.7. Screening for QC Markers with Anti-Tumor Cell Activity Using PLS Analysis

Marker View (AB SCIEX) was used to select, align, and quantify features. The intensity of each ion was normalized to the base peak intensity (BPI) to generate a data matrix that consisted of the RT, *m*/*z* value, and precursor ion intensity. The data matrix file was exported and opened in Excel software. The *m*/*z* data for the isotope were manually filtered in the raw data matrix.

Partial least squares analysis (PLS) was used to find the basic relationship of two matrices (X and Y), a hidden variable method for modeling the covariance structure in these two spaces. The PLS was used to analyze the correlation of the ion intensities of all precursors, with respect to the inhibition rate of tumor cell (SMMC-7721) proliferation of all fractions from Bufonis Venenum samples. The ion intensity is the dependent variable X and the inhibition rate on tumor cells is the independent variable Y. Precursors with VIP value > 1.5 represented high contribution to the anti-tumor cell activities of the corresponding fraction.

### 4.8. Quantitative Determination of 9 High Content Markers in Different Batches of Bufonis Venenum by HPLC-UV

Based on the screening of active markers, we selected 9 bufadienolide standards as quality evaluation indicators for Bufonis Venenum. Samples of Bufonis Venenum from different batches (Sample information is shown in Table 1) and from different production areas were collected. Each sample was ultrasonically extracted with 90% methanol for 1 h and the supernatant was centrifuged at high speed for liquid phase measurement. Quantitative detection of the samples was performed on an L-3000 HPLC system (RIGOL) using an X-Charge column (4.6 mm × 250 mm, 5 µm). A gradient elution of solvent A (0.1% formic acid in water) and solvent B (acetonitrile) was applied as follows: 0–4 min, 20–38% B; 4–20 min, 38% B; 20–30 min, 38–90% B; 30–34 min, 90–90% B; 34–35 min, 90–20% B; 35–45 min, 20% B. The column temperature was 35 °C; volume flow was 0.7 mL/min; and the injection amount was 20 μL, detected at a wavelength of 296 nm.

### 4.9. Methodological Investigation

The quantification method was validated for linearity, LOQs and LODs, repeatability, precision, stability, and accuracy. A mixed standard solution of different concentrations (400 ng/mL, 1000 ng/mL, 2000 ng/mL, 4000 ng/mL, 10,000 ng/mL, 20,000 ng/mL, and 40,000 ng/mL) was prepared according to the HPLC quantitative analysis method injection assay. The linear regression analysis was carried out, using the peak area value as the ordinate (y) with the mass concentration (ng/mL) of the mixed standard solution as the abscissa (x), and the correlation coefficient was calculated.

Intra-day precision and day-to-day precision were measured by repeating the injection of the standard solution six times in one day and for three consecutive days. Precision, repeatability, and stability of the method were also validated for each analyte. In order to confirm the repeatability, 3 batch samples were randomly selected and each sample was prepared in parallel for 3 analyses. The stability of the sample solution was tested at room temperature and the sample solution was analyzed at 0, 2, 4, 6, 12, and 24 h to evaluate stability. Recovery was carried out by adding a known amount of each standard to a certain amount of sample (15mg). The mixture was extracted and analyzed, according to the above method, and the analytical method was shown to be accurate.

### 4.10. Statistical Analysis

PeakerView (AB SCIEX) was used to examine the ion chromatograms (XIC) extracted from the .wiff files from TripleTOF 5600 and MarkerView (AB SCIEX) was used to select, align, and quantify features (chromatographic events corresponding to specific *m*/*z* values and RTs). The intensity of each ion was normalized to the base peak intensity (BPI) to generate a data matrix that consisted of the RT, *m*/*z* value, and marker intensity. The Pearson correlation coefficients of the peak intensities of all precursors, with respect to the inhibition rate of tumor cell (SMMC-7721) proliferation determined in an MTT assay across all of the fractions, were calculated using Microsoft Excel and *p* < 0.05 was considered to be significant and *p* < 0.01 very significant. These data were imported to SIMCA-P for the PLS and PCA analysis.

## Figures and Tables

**Table 1:** The peptides were detected in Bufonis Venenum by LC-MS/ MS (part).

Peptide	Mass	Length	*m*/*z*	Intensity	Protein
FPGNKITSVAGVY	1351.7136	13	676.864	6.52E + 05	CL4590.Contig1_All
ISWLKPS	829.4698	7	415.7423	6.51E + 05	CL4590.Contig1_All
P.R(+44.03)GFPGPPGP.P	924.4817	9	463.2499	1.75E + 04	P39061|COIA1_MOUSE
P.AVPIPLVA(+17.03).P	795.5218	8	398.7651	1.02E + 04	P39061|COIA1_MOUSE
LGDS(+42.01)VVTP	828.4229	8	415.2137	1.68E + 04	CL3802.Contig2_All
S(−20.03)ALPAKV	664.3907	7	333.2047	4.95E + 04	CL3802.Contig2_All
GC(sub R)KRAAKR	888.5076	8	445.2622	3.31E + 05	CL4128.Contig1_All
LPVLGP(sub T)LQLYCA	1285.7104	12	643.8583	1.19E + 04	Unigene3157_All
QPSQR(sub K)R	770.4147	6	386.2119	2.80E + 03	CL5925.Contig2_All
P(sub A)HSNPG	607.2714	6	304.6418	1.40E + 03	Unigene9476_All

**Table 2:** Discovery of chemical markers using PLS analysis.

Var ID	RT *	*m*/*z*	Identification	M1.VIP [2]
(Primary)				
X1	8.29	385.2369	resibufogenin	2.45
X2	6.98	387.2526	bufalin	2.08
X3	4.19	401.2322	hydroxylresibufogenin	2.53
X4	3.29	403.2475	desacetylbufotalin	2.63
X5	4.66	403.248	telocinobufagin	2.41
X6	2.31	403.2481	gamabufotalin	2.35
X7	3.27	415.2114	19-oxo-desacetylcinobufagin	3.06
X8	8.94	415.2126	bufotalinin	2.37
X9	3.15	417.2261	arenobufagin	2.33
X10	2.76	419.2431	hellebrigenol	2.38
X11	2.29	425.231	dehydrated cinobufagin	2.35
X12	8.95	437.1923	unknown	2.44
X13	8.31	443.2421	cinobufagin	2.63
X14	5.12	445.2581	bufotalin	2.97
X15	5.82	459.2356	cinobufaginol	1.71
X16	3.78	641.353	3-(N-succinyl argininyl)- resibufogenin	2.78
X17	1.85	679.5126	unknown	1.59
X18	4.84	685.4183	3-(N-pimeloyl- argininyl)bufalin	1.96
X19	3.46	699.3966	3-(N-suberoyl- argininyl)bufalin	2.66
X20	3.86	713.4094	3-(N-suberoyl argininyl)- desacetylcinobufagin	2.49
X21	2.67	715.3566	3-(N-succinyl- argininyl)gamabufotalin	1.89
X22	2.93	715.4271	3-(N-succinyl argininyl)- telocinobufagin	2.04
X23	2.88	729.4049	3-(N-suberoyl argininyl)- hellebrigenin	2.07
X24	5.1	467.241	unknown	2.92
X25	8.81	374.3625	unknown	2.46
X26	2.31	405.2543	unknown	2.28
X27	3.06	787.412	unknown	2.15
X28	5.97	700.4416	unknown	1.81
X29	0.43	160.0757	Indole-3-acetaldehyde	1.31
X30	0.46	203.1177	dehydrobufotenine	-
X31	0.43	205.1339	bufotenine	-
X32	10.31	221.1169	bufotenine N-oxide	0.36
X33	6.24	256.2635	hexadecanamide	1.01
X34	0.39	275.1354	Indole-3-acetyl-L-valine	0.85
X35	7.14	331.1964	glycerol 1-monopalmitate	-
X36	7.75	335.2014	Strychnine	-

* RT: retention time.

**Table 3:** Calibration curves, LOD and LOQ for 9 bufadienolides.

Substance Name	Standard Curve Line	r^2^	Linear Range (ng/mL)	LOD (ng/mL)	LOQ (ng/mL)
gamabufotalin	y = 0.0134x − 0.8377	0.9999	400–40,000	241.93	660.56
arenobufagin	y = 0.0216x − 1.9421	0.9999	400–40,000	230.98	560.12
telocinobufagin	y = 0.0158x − 0.8980	0.9998	400–40,000	356.21	1054.75
desacety-bufotalin	y = 0.0279x − 4.7703	0.9998	400–40,000	347.39	759.02
bufotalin	y = 0.0188x − 2.9766	0.9999	400–40,000	458.70	1159.55
cinobufotalin	y = 0.0204x − 3.5772	0.9997	400–40,000	491.72	1229.93
bufalin	y = 0.0215x − 1.9680	0.9999	400–40,000	231.90	559.43
resibufogenin	y = 0.0219x − 3.5778	0.9999	400–40,000	259.16	482.55
cinobufagin	y = 0.0122x − 1.1549	0.9999	400–40,000	275.48	697.37

**Table 4:** Precision, repeatability, stability, and recovery for 9 bufadienolides.

Substance Name	Precision RSD (%)		Repeatability RSD (%)	Stability RSD (%)	Recovery (%)	
	Intraday	Interday			RSD (%)	Mean (%)
gamabufotalin	2.8	1.6	0.7	0.7	1.5	97.0
arenobufagin	2.6	2.2	0.8	1.7	0.8	103.4
telocinobufagin	2.5	3.6	1.3	1.6	1.4	91.1
desacety-bufotalin	3.2	3.4	3.2	4.5	2.9	92.2
bufotalin	2.5	4.9	0.9	2.9	1.0	105.3
cinobufotalin	5.7	4.6	1.6	2.1	0.8	104.1
bufalin	2.3	2.7	0.9	1.1	1.2	101.8
resibufogenin	2.8	1.6	1.0	1.4	0.8	102.5
cinobufagin	4.9	3.8	0.6	1.3	0.7	103.1

**Table 5:** Bufonis Venenum samples from different Origins.

	gamabu-fotalin	arenob-ufagin	telocino-bufagin	desacety-bufotalin	bufotalin	cinobu-fotalin	bufalin	resibufo-genin	cinobu-fagin	Inhibition Rate (%)
Shandong-1	1.7	3.41	0.29	0.01	1.04	0.60	1.29	4.41	2.22	52.05 ± 0.58
Shandong-2	1.3	3.80	0.38	0.02	1.05	0.88	1.22	3.21	2.41	53.79 ± 0.96
Shandong-3	1.67	3.32	0.28	0.01	1.01	0.54	1.15	3.79	1.82	52.17 ± 0.64
Linyi-1	0.89	1.46	1.08	0.16	1.16	1.86	1.40	1.84	3.68	50.49 ± 0.36
Linyi-2	0.97	1.52	1.32	0.12	1.21	1.76	1.33	1.65	3.30	50.1 ± 0.30
Nantong-1	0.69	3.59	0.89	0.09	1.60	1.81	1.68	1.80	3.86	55.04 ± 1.00
Nantong-2	0.75	2.60	0.89	0.17	1.49	2.02	1.61	2.02	4.46	56.29 ± 0.80
Nantong-3	0.79	1.57	0.41	0.17	1.72	1.64	2.53	2.81	4.86	52.84 ± 1.60
Nantong-4	0.64	1.64	1.81	0.12	1.41	2.21	1.81	2.09	4.04	53.69 ± 1.22
Nantong-5	0.46	3.41	1.28	0.07	1.33	2.19	1.36	1.21	2.72	55.66 ± 0.94
Xuzhou-1	0.59	1.30	0.74	0.1	1.33	2.11	1.55	2.41	3.70	54.31 ± 1.27
Xuzhou-2	1.11	1.31	1.3	0.21	1.41	2.12	1.55	2.42	3.72	51.97 ± 1.44
Sanyitang-2	1.08	4.20	1.9	0.13	1.46	2.00	1.70	1.77	4.19	60.71 ± 1.45
Sanyitang-5	1.06	3.69	1.61	0.34	1.39	2.22	1.61	1.57	3.58	59.47 ± 0.93
Sanyitang-7	1.02	3.33	0.62	0.31	1.32	1.68	1.40	1.39	2.64	56.92 ± 1.48
Sanyitang-8	0.84	2.06	1.38	0.11	0.99	0.98	1.56	2.68	3.63	55.59 ± 1.01
Sanyitang-9	0.94	0.72	1.15	0.24	0.67	0.89	1.66	4.10	4.48	53.06 ± 1.07
Sanyitang-10	1.15	1.40	0.91	0.36	1.64	2.23	1.38	1.65	3.14	55.18 ± 3.18
Sanyitang-11	0.5	3.15	1.01	0.1	1.23	1.68	1.44	1.49	3.28	58.32 ± 1.76
Sanyitang-12	0.88	1.89	1.07	0.22	1.02	1.26	1.30	1.91	3.02	54.79 ± 1.64
Taiji-1	1.15	3.32	1.21	0.13	1.04	1.43	1.33	1.80	3.42	59.01 ± 2.88
Taiji-2	1.13	3.64	1.18	0.12	1.18	1.55	1.46	1.99	3.75	53.55 ± 0.95
Taiji-3	0.96	2.92	1.44	1.3	1.14	1.45	1.23	1.25	2.71	52.05 ± 0.80
Taiji-4	0.93	1.31	1.32	0.12	1.59	2.27	1.53	1.89	3.56	51.37 ± 0.39
leiyunshang-1	0.26	0.45	0.27	0.02	1.64	0.35	2.48	7.08	4.53	51.31 ± 0.27
leiyunshang-3	0.28	0.38	0.29	0.03	1.68	0.25	2.64	7.12	6.29	54.53 ± 1.30
leiyunshang-4	0.33	0.42	0.26	0.06	1.33	0.11	2.49	8.99	4.11	51.31 ± 0.28
leiyunshang-5	0.33	0.64	0.31	0.05	1.81	0.48	2.26	5.84	5.59	55.72 ± 0.44
leiyunshang-6	0.37	0.43	0.33	0.06	1.76	0.14	2.72	7.37	5.85	56.56 ± 1.28
Correlation coefficient	−0.07	0.40	0.28	−0.04	0.13	0.20	−0.02	−0.24	0.12	

## Supplementary Materials

The following are available online, Table S1: Structure of the main compounds in Bufonis Venenum, Table S2: Bufonis Venenum samples from different Origins, Table. S3*. Standardized value of different cell inhibition rates, Table S4. Standardized value of correlation coefficient for PLS analysis.

## References

[1] Kang L.P., Zhao Y., Pang X., Yu H.S., Xiong C.Q., Zhang J., Gao Y., Yu K., Liu C., Ma B.P. (2013). Characterization and identification of steroidal saponins from the seeds of Trigonella foenum-graecum by ultra high-performance liquid chromatography and hybrid time-of-flight mass spectrometry. J. Pharm. Biomed. Anal..

[2] Zhang X.X., Liang J.R., Liu J.L., Zhao Y., Gao J., Sun W.J., Ito Y. (2014). Quality control and identification of steroid saponins from Dioscorea zingiberensis C. H. Wright by fingerprint with HPLC-ELSD and HPLC-ESI-Quadrupole/Time-of-fight tandem mass spectrometry. J. Pharm. Biomed. Anal..

[3] Li S.L., Shen H., Zhu L.Y., Xu J., Jia X.B., Zhang H.M., Lin G., Cai H., Cai B.C., Chen S.L. (2012). Ultra-high-performance liquid chromatography–quadrupole/time of flight mass spectrometry based chemical profiling approach to rapidly reveal chemical transformation of sulfur-fumigated medicinal herbs, a case study on white ginseng. J. Chromatogr. A.

[4] Zhu J.B., Guo X.J., Fu S.P., Zhang X.L., Liang X.M. (2010). Characterization of steroidal saponins in crude extracts from Dioscorea zingiberensis C. H. Wright by ultra-performance liquid chromatography/electrospray ionization quadrupole time-of-flight tandem mass spectrometry. J. Pharm. Biomed. Anal..

[5] Lin S.H., Wang D.M., Yang D.P., Yao J.H., Tong Y., Chen J.P. (2007). Characterization of steroidal saponins in crude extract from Dioscorea nipponica Makino by liquid chromatography tandem multi-stage mass spectrometry. Anal. Chim. Acta.

[6] Wu K.C., Kao C.P., Ho Y.L., Chang Y.S. (2017). Quality Control of the Root and Rhizome of Helminthostachys zeylanica (Daodi-Ugon) by HPLC Using Quercetin and Ugonins as Markers. Molecules.

[7] Wei F.H., Chen M.T., Luo C.H., Chen F.L., Shen Q., Mo Z.X. (2016). Developing an Absorption-Based Quality Control Method for Hu-Gan-Kang-Yuan Capsules by UFLC-QTOF-MS/MS Screening and HPLC-DAD Quantitative Determination. Molecules.

[8] Yin T.J., Yang G.Y., Ma Y., Xu B.B., Hu M., You M., Gao S. (2015). Developing an activity and absorption-based quality control platform for Chinese traditional medicine: Application to Zeng-Sheng-Ping (Antitumor B). J. Ethnopharmacol..

[9] Hu Y.H., Jiang P., Wang S.P., Yan S.K., Xiang L., Zhang W.D., Liu R.H. (2012). Plasma pharmacochemistry based approach to screening potential bioactive components in Huang-Lian-Jie-Du-Tang using high performance liquid chromatography coupled with mass spectrometric detection. J. Ethnopharmacol..

[10] Lucio-Gutierrez J.R., Garza-Juarez A., Coello J., Maspoch S., Salazar-Cavazos M.L., Salazar-Aranda R., Waksman de Torres N. (2012). Multi-wavelength high-performance liquid chromatographic fingerprints and chemometrics to predict the antioxidant activity of Turnera diffusa as part of its quality control. J. Chromatogr. A.

[11] Ren Y.S., Zhang P., Yan D., Wang J.B., Du X.X., Xiao X.H. (2011). A strategy for the detection of quality fluctuation of a Chinese herbal injection based on chemical fingerprinting combined with biological fingerprinting. J. Pharm. Biomed. Anal..

[12] Yang E., Zhong Y.M., Feng Y.F. (2012). Advance on the chemical constituents and pharmacological effects of Atractylodes macrocephala Koidz. J. Guangdong Pharm. Univ..

[13] Yin H., Yin H., Wang Z.Q., Wang L., Zhou A.Z., Li Q.L., Cheng Z. (2013). Simultaneous determination of Atractylone, Atractylenolide I, II, III in Atractylodes macrocephala by HPLC-wavelength switching method. Chin. J. Tradit. Chin. Med..

[14] Tang D.D., Yuan S.J., Zhang N., Zhao Y.H., Wei F., Zhao Y.Y. (2016). Research progress of pharmacodynamics and toxicity of traditional Chinese medicine based on urinary metabolomics. Chin. J. Pharm. Anal..

[15] Lu C.M., Cui X.M. (2019). Research Progress in TCM Quality Control and Toxicity Evaluation Based on Metabolomics Technology. Chin. J. Inf. Tradit. Chin. Med..

[16] Koek M.M., Jellema R.H., Greef. J., Tas A.C., Hankemeier T. (2011). Quantitative metabolomics based on gas chromatography mass spectrometry: Status and perspectives. Metabolomics.

[17] Pellati F., Orlandini G., Benvenuti S. (2012). Simultaneous metabolite fingerprinting of hydrophilic and lipophilic compounds in Echinacea pallida by high-performance liquid chromatography with diode array and electrospray ionization-mass spectrometry detection. J. Chromatogr. A..

[18] Kim E.J., Kwon J., Park S.H., Park C., Seo Y.B., Shin H.K., Kim H.K., Lee K.S., Choi S.Y., Ryu D.H. (2011). Metabolite profiling of Angelica gigas from different geographical origins using 1H NMR and UPLC-MS analyses. J. Agric. Food Chem..

[19] Zhang P., Cui Z., Liu Y.S., Wang D., Liu N., Yoshikawa M. (2005). Quality evaluation of traditional Chinese drug toad venom from different origins through a simultaneous determination of bufogenins and indole alkaloids by HPLC. Chem. Pharm. Bull..

[20] Gao H.M., Zehl M., Leitner A., Wu X.Y., Wang Z.M., Kopp B. (2010). Comparison of toad venoms from different Bufo species by HPLC and LC-DAD-MS/MS. J. Ethnopharmacol..

[21] Sun C.F., Fan S.C., Luo Y. (2018). Research progress on chemical constituents and artificial synthesis of Bufonis Venenum. Chin Tradit. Herbal Drugs.

[22] Wang Z.Y., Zhou J., Ma H.Y., Zhu Z.H., Qian D.W., Duan J.A., Wu Q.N. (2017). Identification of Proteins in Toad Venom by NanoLC-LTQ-Orbitrap Velos Pro. Chin. Pharm. J..

[23] Huo Y.G., Xv R.X., Ma H.Y., Zhou J., Xi X.P., Wu Q.N., Duan J.A., Zhou M., Chen T.B. (2018). Identification of < 10 KD peptides in the water extraction of Venenum Bufonis from Bufo gargarizans using Nano LC– MS/MS and De novo sequencing. J. Pharm. Biomed. Anal..

[24] Chen Y.L., Hao Y.Y., Guo F.J. (2017). Research progress on chemical constituents and pharmacological activities of Bufonis Venenum. Chin. Tradit. Herbal Drugs.

[25] Gao X.X., Sun C.P., Yu Z.L., Jian C., Tian X.G., Huo X.K., Lei F., Liu X.G., Wang C., Zhang B.J. (2018). Correlation analysis between the chemical contents and bioactivity for the quality control of Alismatis Rhizoma. Acta Pharm. Sin. B.

[26] Xu H.Y., Hou W.B., Li. K. (2016). A new concept on quality marker of Chinese materia medica: Quality control for Chinese medicinal products. Chin. Tradit. Herbal Drugs.

[27] Liu C.X. (2016). Recognizing healthy development of Chinese medicine industry from resourcesquality-quality markers of Chinese medicine. Chin. Tradit. Herbal Drugs.

[28] Wang J. (2009). Study on the Relationship of the Structure-Effect-Toxicity of Bufadienolides Inhibiting Tumor Cells Proliferation. Master’s Thesis.

[29] Liu D., Zhang W., Wang X.Y., Chen T., Hu W. (2018). Treatment of Cionbufagin on analgesic effect and activation of glial cells in the spinal cord of cancer-induced bone pain rats. Carcinog. Teratog. Mutagen..

[30] Cai H., Xu Y.Y., Xie L., Duan Y., Zhou J., Liu J., Niu M.J., Zhang Y.T., Shen L., Pei K. (2019). Investigation on Spectrum-Effect Correlation between Constituents Absorbed into Blood and Bioactivities of Baizhu Shaoyao San before and after Processing on Ulcerative Colitis Rats by UHPLC/Q-TOF-MS/MS Coupled with Gray Correlation Analysis. Molecules.

