# Supplementary material for: High Resolution Mass Profile of Bufadienolides and Peptides Combing with Anti-Tumor Cell Screening and Multivariate Analysis for the Quality Evaluation of Bufonis Venenum
Source: Molecules. 2019 May 20;24(10):1943. doi: 10.3390/molecules24101943 (PMC6572007; doi:10.3390/molecules24101943)
Supplement: Supplementary file 1 [file molecules-24-01943-s001.pdf]

molecules

MDPI

High Resolution Mass Profile of Bufadienolides and Peptides Combing with Anti-Tumor Cell Screening and Multivariate Analysis for the Quality Evaluation of Bufonis Venenum

Rongrong He [1], Hongyue Ma [1,*], Jing Zhou [1,*], Zhenhua Zhu [1], Xiang Lv [1], Quan Li [2], Hengbin Wang [2], Yanqing Yan [2], Niancui Luo [2], Liuqing Di [1], Qinan Wu [1] and Jinao Duan [1]

Supplementary Materials:

Table. S1. Structure of the main compounds in Bufonis Venenum.

Compound	Skeletons	R	n
bufalin	A1	R_1=R_3=H, R_2=CH3, R_5=OH, R_4=R_6=R_7=R_8=R_9=H	-
gamabufotalin	A1	R_1=R_3=R_4=R_7=R_8=R_9=H, R_2=CH_3, R_5=OH, R_6=α-OH	-
bufotalin	A1	R_1=R_3=R_4=R_6=R_7=R_9=H, R_2=CH_3, R_5=OH, R_8=β-OAc	-
hellebrigenin	A1	R_1=R_3=R_6=R_7=R_8=R_9=H, R_2=CHO, R_4=R_5=OH	-
telocinobufagin	A1	R_1=R_3=R_6=R_7=R_8=R_9=H, R_2=CH_3, R_4=R_5=OH	-
resibufogenin	A2	R_1=R_3=R_4=R_5=R_6=R_7=R_8=H, R_2=CH_3	-
resibufagin	A2	R_1=R_3=R_4=R_5=R_6=R_7=R_8=H, R_2=CHO	-
3β-formyloxylresibufogenin	A2	R_1=HCO, R3=R4=R5=R6=R7=R8=H, R2=CH3	-
12β-hydroxyresibufaginol	A2	R_1=R3=R4=R5=R6=R8=H, R2=CH3, R7=β-OH	-
resibufaginol	A2	R_1=R3=R4=R5=R6=R7=R8=H, R2=CH2OH	-
cinobufagin	A2	R_1=R_3=R_4=R_5=R_6=R_7=H, R_2=CH_3, R_8=β-OAc	-
desacetylcinobufagin	A2	R_1=R_3=R_4=R_5=R_6=R_7=H, R_2=CH_3, R_8=β-OH	-
cinobufotalin	A2	R_1=R_3=R_5=R_6=R_7=H, R_2=CH_3, R_4=OH, R_8=β-OAc	-
desacetylcinobufotalin	A2	R_1=R_3=R_5=R_6=R_7=H, R_4=OH, R_2=CH_3, R_8=β-OH	-
arenobufagin	A3	R_1=R_2=H	-
psi-bufarenogin	A4	R_1=α-OH, R2=H	-
bufarenogin	A4	R_1=β-OH, R2=H	-
20S,21-epoxyresibufogenin	A5	R_1=OH	-
20R,21-epoxyresibufogenin	A5	R_1=OH	-
3-O-formyl-20R,21-epoxyresibufogenin	A5	R_1=HCO	-
3-(N-succinyl argininyl) resibufogenin	B	R_1= CH_3, R_2=R_3=H	2
3-(N-pimeloyl argininyl)bufalin	B	R_1= CH_3, R_2=R_3=R_4=R_5=H	5
3-(N-suberoyl argininyl)bufalin	B	R_1= CH_3, R_2=H, R_3=OAc	2
3-(N-suberoyl argininyl) desacetylcinobufagin	B	R_1= CH_3, R_2=H, R_3=OH	6
3-(N-succinyl argininyl)gamabufotalin	B	R_1=CH_2OH, R_2=H, R_3=OAc	2
3-(N-succinyl argininyl) telocinobufagin	B	R_1= CH_3, R_2=OH, R_3=R_4=R_5=H	6
3-(N-suberoyl argininyl) hellebrigenin	B	R_1=CHO, R_2=OH, R_3=R_4=R_5=H	6
5-hydroxytryptamine	C	R_1=OH, R_2=NH_2	-
bufotenine	C	R_1=OH, R_2=$N(CH_3)_2$	-
bufotenine N-oxide	C	R_1=OH, R_2=$NOCH_3$	-
bufobutanoic acid	C	R_1=OH, R_2= NH $CO(CH_2)_2COOH$	-
bufotenidine	C	R_1=O^-, R_2= $N(CH_3)^{3+}$	-
cholesterol	D	-	-

Table. S2: Bufonis Venenum samples from different Origins

Sample No.	Name	Date	Location
1	Shandong-1	Oct, 2017	Shandong
2	Shandong-2	Apr, 2017	Shandong
3	Shandong-3	May, 2017	Shandong
4	Linyi-1	May, 2017	Linyi, Shandong
5	Linyi-2	Jan, 2017	Linyi, Shandong
6	Nantong-1	Oct, 2014	Nantong, Jiangsu
7	Nantong-2	June, 2015	Nantong, Jiangsu
8	Nantong-3	Aug, 2015	Nantong, Jiangsu
9	Nantong-4	Mar, 2016	Nantong, Jiangsu
10	Nantong-5	Feb, 2017	Nantong, Jiangsu
11	Xuzhou-1	Jan, 2017	Sanyitang, Anhui
12	Xuzhou-2	Jan, 2017	Sanyitang, Anhui
13	Sanyiyang-1	Jan, 2017	Sanyitang, Anhui
14	Sanyiyang-2	Jan, 2017	Sanyitang, Anhui
15	Sanyiyang-3	Jan, 2017	Sanyitang, Anhui
16	Sanyiyang-4	Jan, 2017	Sanyitang, Anhui
17	Sanyiyang-5	Jan, 2017	Sanyitang, Anhui
18	Sanyiyang-6	Jan, 2017	Sanyitang, Anhui
19	Sanyiyang-7	May, 2017	Sanyitang, Anhui
20	Sanyiyang-8	Jan, 2017	Taiji, Chongqing
21	Taiji-1	Jan, 2017	Taiji, Chongqing
22	Taiji-2	Jan, 2017	Xuzhou, Jiangsu
23	Taiji-3	Jan, 2017	Xuzhou, Jiangsu
24	Taiji-4	May, 2017	Xuzhou, Jiangsu
25	Leiyunshang-1	Aug, 2018	Suzhou, Jiangsu
26	Leiyunshang-2	Aug, 2018	Suzhou, Jiangsu
27	Leiyunshang-3	Aug, 2018	Suzhou, Jiangsu
28	Leiyunshang-4	Aug, 2018	Suzhou, Jiangsu
29	Leiyunshang-5	Aug, 2018	Suzhou, Jiangsu

Table. S3*. Standardized value of different cell inhibition rates

	Hela	Tca-8113	SK-OV-3	mean	x_i*
resibufogenin	16.37	11.02	10.48	12.63	0.2998
cinobufagin	43.11	60.39	40.15	47.88	1.1368
bufalin	63.38	62.07	48.47	57.97	1.3764
bufotalin	65.38	56.79	36.89	53.02	1.2588
gamabufotalin	42.15	28.91	32.01	34.36	0.8157
arenobufagin	61.19	56.82	36.43	51.48	1.2222
desacety-bufotalin	45.93	51.60	32.85	43.46	1.0318
telocinobufagin	38.82	54.44	30.60	41.29	0.9802
cinobufotalin	44.55	39.95	26.51	37.00	0.8785

Table. S3*: Quoted from the experimental data of Wang Jian, a student guided by Professor Ma Hongyue of the research group. x_i*: The standardized value of the inhibition rates of nine bufadienolides on different cells.

Table S4. Standardized value of correlation coefficient for PLS analysis.

	**VIP value**	y_i*
resibufogenin	2.45	1.023
cinobufagin	2.63	1.098
bufalin	2.08	0.868
bufotalin	2.97	1.240
gamabufotalin	2.35	0.981
arenobufagin	2.33	0.972
desacety-bufotalin	2.63	1.098
telocinobufagin	2.41	1.006
cinobufotalin	1.71	0.714

y_i*: The standardized value of the VIP value of the PLS analysis
